# Glycan Modifications as Regulators of Stem Cell Fate

**DOI:** 10.3390/biology13020076

**Published:** 2024-01-26

**Authors:** Raghad Alghazali, Ahmed Nugud, Ahmed El-Serafi

**Affiliations:** 1 Department of Biomedical and Clinical Sciences (BKV), Linköping University, 58183 Linköping, Sweden; raghad.alghazali@icloud.com; 2 Clinical Sciences, University of Edinburgh, Edinburgh EH4 2XU, UK; s2004480@ed.ac.uk; 3 Gastroenterology, Hepatology & Nutrition, Sheikh Khalifa Medical City, Abu Dhabi 51900, United Arab Emirates; 4 Department of Hand Surgery, Plastic Surgery and Burns, Linköping University, 58185 Linköping, Sweden

**Keywords:** glycosylation, synthetic glycans, stem cell differentiation

## Abstract

**Simple Summary:**

Stem cells represent hope for millions of patients seeking prompt recovery. Unfortunately, the process of converting stem cells into the target cells that will replace the failed or lost organ is still incompletely efficient. One of the underestimated factors that can affect this process is the complex sugar content on the cell surface or in the surrounding environment. In this article, we briefly reviewed the main types of sugars added to the surface of cell proteins, followed by a reflection on their role in stem cells at their original state and during their transformation to a specialized cell type, such as the cells of bones, heart, brain, etc. By the end, we explained different strategies that can be used to increase the efficiency of this process by adding certain types of sugars to the environment around the cells or a three-dimensional composite. Understanding the role of added sugars in the process of stem cell differentiation can provide another clue, ultimately advancing the field of regenerative medicine.

**Abstract:**

Glycosylation is a process where proteins or lipids are modified with glycans. The presence of glycans determines the structure, stability, and localization of glycoproteins, thereby impacting various biological processes, including embryogenesis, intercellular communication, and disease progression. Glycans can influence stem cell behavior by modulating signaling molecules that govern the critical aspects of self-renewal and differentiation. Furthermore, being located at the cell surface, glycans are utilized as markers for stem cell pluripotency and differentiation state determination. This review aims to provide a comprehensive overview of the current literature, focusing on the effect of glycans on stem cells with a reflection on the application of synthetic glycans in directing stem cell differentiation. Additionally, this review will serve as a primer for researchers seeking a deeper understanding of how synthetic glycans can be used to control stem cell differentiation, which may help establish new approaches to guide stem cell differentiation into specific lineages. Ultimately, this knowledge can facilitate the identification of efficient strategies for advancing stem cell-based therapeutic interventions.

## 1. Introduction

Glycans are ubiquitous sugar molecules on the outer surface of all cells in nature and serve as essential markers for the identification and isolation of distinct cell types [1]. Beyond their role as markers, the complexity of glycans is multifaceted, as their structures are not only unique at every level of biological organization—from species level down to individual molecules—but they also exhibit dynamic changes throughout development and disease [2,3]. The complex biosynthesis of glycans further contributes to their enigmatic nature, as they are not directly encoded within the genome. Instead, these compounds are synthesized in correspondence to the activity of glycosidases and glycosyltransferases on the cytosolic and luminal faces of the Endoplasmic Reticulum (ER) and within the Golgi apparatus (GA) [4,5]. There are over 300 identified human glycosyltransferases and glycosidases, and their expression and activity are influenced by internal and external factors [6,7]. Nearly all cell surface proteins undergo glycosylation, with approximately 50% of glycosylated proteins being secreted [4]. These protein-bound glycans decisively govern the structure, stability, and localization of glycoproteins, thereby holding paramount importance in biological processes such as protein folding and quality control. The presence or absence of glycans exerts significant influence over an array of biological processes, encompassing development, tumorigenesis, and inflammation [8]. In many cases, specific functions of glycans remain elusive, and the same glycan may serve different functions based on the type of aglycone (protein or lipid) to which it attaches [9]. In multicellular organisms, glycan components of matrix molecules, including proteoglycans, are pivotal for maintaining tissue structure, porosity, and integrity [9]. Thick layers of glycans serve as a crucial physical protective barrier. For instance, the dense layer of mucins coating many epithelial surfaces, present in the inner linings of airways and intestines, provides protection against pathogen invasion [10,11]. Certain glycans can also act as a storage depot for biologically important molecules. Hydrophilic glycans on cell surfaces and extracellular matrices can serve as a depot for water molecules [12], while extracellular matrix glycosaminoglycans and polysialic acid can locally store growth factors and other bioactive molecules and release them as needed, particularly during processes like injury and wound healing [13,14,15]. Furthermore, glycans play an essential role as mediators of cell–cell interactions, cell–extracellular matrix interactions, and, most notably, interactions between ligands and receptors. Examples include Wnt receptor, fibroblast growth factor (FGF) receptor, Hedgehog (Hh) receptor, and bone morphogenetic protein (BMP) receptor interactions [1].

Notably, genetic mutations linked to glycosylation processes have been pinpointed in several inherited disorders, collectively referred to as congenital disorders of glycosylation [16]. Furthermore, cell surface glycans regulate immune responses, inflammatory reactions, and host–pathogen recognition, as pathogens often exploit specific sialic acid linkages to facilitate their entry into host cells [17,18,19]. Remarkably, dysregulated glycosylation machinery is associated with tumor development and progression, where the aberrant glycome of tumors is thought to explain the heterogeneity seen in numerous cancers [20,21]. The implications of glycosylation in cancer and cancer stem cells have been comprehensively reviewed in the other literature and are not considered to be the focus of this article [21,22]. Given the multitude of roles that glycans play in maintaining distinct biological functions, it is unsurprising that glycans are regarded as universal in their nature as other major macromolecular building blocks (nucleic acids, proteins, and lipids), and as indispensable for the existence of all known living organisms [13,23,24].

Stem cells are attracting considerable attention due to their ability to differentiate and regenerate lost or damaged tissues. Despite decades of research, harnessing the differentiation of stem cells is still a target to be achieved, while the role of glycans in this process is often underestimated. Interestingly, glycans are pivotal for modulating signaling molecules that govern self-renewal and differentiation [25]. Glycans have proven particularly valuable as markers for discerning the pluripotent status of mouse embryonic stem cells and human induced pluripotent stem cells due to their presence on the cell surface. For example, many well-known stem cell markers, such as stage-specific embryonic antigen-1 (SSEA-1), SSEA-3, SSEA-5, as well as the tumor-rejection antigens (TRA)-1–60 and 1–81, are composed of glycans [26,27,28]. Additionally, there is growing evidence suggesting that glycans play a role in maintaining stem cell pluripotency [1,29]. Furthermore, glycans offer a distinctive opportunity for steering or manipulating stem cell differentiation. Innovative strategies in cell surface engineering have emerged, providing opportunities to control stem cell differentiation. These strategies encompass chemoenzymatic methods for editing existing cell surface glycan structures [30], as well as metabolic approaches to introduce non-natural monosaccharide modifications across the glycome [31]. This review aims to provide an overview of the current literature focusing on the role of glycosylation in stem cell differentiation and fate decision, with special emphasis on the emerging utility of synthetic glycans in directing stem cell differentiation toward distinct cell lineages.

## 2. Glycosylation

Glycosylation is an ubiquitous and indispensable co- and/or post-translational modification required for the normal biological functioning of cells. Glycosylation occurring midway through folding, significantly contributes to the accurate three-dimensional structure of the protein [32]. To date, several types of protein glycosylation have been identified, each characterized by unique protein–sugar linkages [33].

### 2.1. Glycosylation Types

#### 2.1.1. N-Linked Glycosylation

The process of N-glycosylation involves attaching N-acetylglucosamine (GlcNAc) to the nitrogen atom of an asparagine (Asn) side chain through a β–1N linkage [34]. Adding or removing various monosaccharides like galactose, results in different glycan structures categorized as high-mannose N-glycans, complex N-glycans, or hybrid N-glycans. The dynamic process of N-linked glycosylation plays a crucial role in determining the diversity and functionality of glycoproteins, with significant implications in cellular physiology [9].

#### 2.1.2. O-Linked Glycosylation

O-Glycosylation involves the attachment of sugar molecules, predominantly N-acetylgalactosamine (GalNAc), as well as other monosaccharides to specific oxygen atoms of serine (Ser) or threonine (Thr) residues within a polypeptide chain [35]. GalNAc-linked glycans, which are also known as mucin-type O-glycans, are found abundantly in various extracellular mucin secretions on mucosal surfaces [35].

### 2.2. Glycosaminoglycan Modifications

Glycosaminoglycans (GAGs) are linear polymers of repeating disaccharide units that form the extracellular matrix and play a crucial role in cell signaling and adhesion [36]. Four major classes of GAGs have been identified, including hyaluronan, heparan sulfate (HS), chondroitin sulfate/dermatan sulfate, and keratan sulfate [4]. Hyaluronan (HA) is a versatile glycosaminoglycan polymer and a fundamental component of the extracellular matrix in several tissues. HA comprises repeating units of glucuronic acid (GlcA) and N-acetylglucosamine (GlcNAc) [37]. HA has various chain lengths, thus influencing various biological functions and physiological roles [38]. Heparan sulfate (HS) proteoglycans are present on cell surfaces, as well as within the basement membrane, and play a pivotal role in cellular growth and differentiation [39]. Many growth factors and cytokines recognize specific HS present on cell surface proteoglycans, which act as co-receptors for the signaling molecules. The sulfation arrangement within HS chains profoundly influences the specificity of interaction [39,40]. Chondroitin sulfate (CS) and its isomeric variant, dermatan sulfate (DS), are major components of the extracellular matrix in various tissues [41]. CS and DS are known to serve as ligands for a variety of growth factors and regulate many cellular events including morphogenesis, proliferation, and differentiation [42]. Keratan sulfate (KS) is present in the cornea, as well as central and peripheral nervous systems [43]. While the focus on KS has historically centered around its sulfated regions, potential functional roles for the poly-N-acetyllactosamine regions of KS have also been described [44].

### 2.3. Sialylation

Sialylation, a vital terminal modification of complex carbohydrates, involves the addition of sialic acid residues to the non-reducing ends of mature N- and O-linked glycans [45]. In addition to providing a negative charge and hydrophilicity to cell surfaces, sialic acids function as receptors for pathogens and toxins [46,47]. Sialylation also plays a crucial role in cell adhesion and regulates the biological stability of glycoproteins [25,48]. Sialylated glycans play a crucial role in mammalian development by coating the cell surface and orchestrating an array of biological activities by masking sub-terminal galactose residues from receptor recognition [4]. Without the development of this coat of sialylated glycans, abnormal cellular development, and maturation may occur [25,45,49]. Sialic acids, unlike other sugars, have a unique capacity to form homo-oligomers or polymers, including disialic acid, oligosialic acid, and polysialic acid [45].

## 3. Glycosylation Effect on Stem Cells and Their Differentiation

The link between cell surface glycans and the status of stem cells, whether in their naïve status or throughout the differentiation process, has been extensively explored (Table 1). The change of glycome signature was studied in accordance with the lineage of interests. While comprehensive global characterization is still warranted, examples highlighting alterations in glycosylation patterns of different proteins and their impact on stem cells and their differentiation are summarized in Table 2. Furthermore, the role of glycans in epigenetics is well established. The addition of O-GlcNAc residues to histone proteins is a key component of the histone code that regulates gene expression. O-GlcNAcylation targets key transcriptional and epigenetic regulators including RNA polymerase II, histones, histone deacetylase complexes, and members of the Polycomb and Trithorax groups. As O-GlcNAc cycling relies on cytosolic UDP–N-acetyl–glucosamine (UDP–GlcNAc) levels, it is considered a homeostatic mechanism linking nutrient availability to higher-order chromatin organization [50,51,52]. Evidence also suggests that this glycosylation mechanism can also influence X chromosome inactivation and genetic imprinting, given that the O-GlcNAc transferase is encoded on the X chromosome [13]. Our group has previously shown the effect of histone modification on the differentiation of stem cells into the chondrogenic and adipogenic lineages as well as to insulin-secreting cells [53,54,55]. Unfortunately, the glycosylation effect has not been investigated in those studies.

### 3.1. Naïve Stem Cells

The dynamics of glycosylation change depending on the stage of embryonic stem cells (ESC). For example, the expression of O-glycosyltransferase increases after ESC formation of embryoid bodies (EBs) or differentiation into endoderm cells, suggesting a pivotal role of O-glycosylation in early commitment [56]. Furthermore, O-glycosylation is instrumental in maintaining the epithelial state of trophoblast stem cells, which are derived from the first embryonic lineage commitment [57].

Sialyation also plays a significant role in maintaining the stem cell population within the body. Deficient sialyation, on the other hand, leads to depletion of stem cell progenitors [3,58]. Notably, the sialic acid linkage undergoes significant changes as cells transition from pluripotency to differentiated progenitors. For instance, human pluripotent stem cells exhibit high levels of surface α2–6-linked sialic acid, whereas the progenitors and terminally differentiated somatic cells present α2–3-linked sialic acid on their surface [29,59]. Interestingly, the reprogramming of human dermal fibroblasts into induced pluripotent stem cells (iPSC) reinstates the α2–6-linked form of sialic acids [29].

In a study by Wang et al. (2015), a significant shift in protein sialylation levels was reported during differentiation. The levels of ST6GAL1 sialyltransferase were significantly elevated in undifferentiated human pluripotent stem cells compared to their non-pluripotent counterparts. Furthermore, the efficiency of somatic cell reprogramming diminished in response to *St6Gal1* gene knockdown and the presence of a sialyltransferase inhibitor. Inhibiting ST6GAL1 in human pluripotent stem cells also resulted in a downregulation of OCT4 protein levels and induced alterations in the expression of various genes associated with cell morphogenesis during differentiation [60]. In contrast, hyperexpression of α2,6–sialyltransferase was associated with pluripotent stem cell lineage commitment and the reduction of their potency [3,58,61,62,63]. Other studies demonstrated that enzymatic removal of sialic acid from the cell surface induced the differentiation of iPSC generated from menstrual blood-derived mesenchymal cells towards the ectodermal lineage and resulted in high amounts of terminal β–galactopyranoside residues, which are potentially sensed by cell surface carbohydrate-binding proteins to induce spontaneous differentiation [64]. Additional studies reported significantly higher levels of α–2–6–sialylated glycan in differentiated cells and a marked reduction in α–2–6–sialylation in highly undifferentiated cells [3,4,45,65].

Moreover, Tateno et al. (2016) conducted a study utilizing a high-density lectin microarray to compare the glycome of early passages of adipose-derived hMSCs with that of cells from late passages, which exhibited a marked reduction in their differentiation efficiency. Four α2–6 Sia-specific lectins (TJA1, SSA, SNA, and rPSL1a) showed the most significant differences in signal intensity between early and late passages of adipose-derived hMSCs, bone marrow-derived hMSCs, and cartilage tissue-derived chondrocytes. These findings suggest that the binding of α2–6 Sia-specific lectins may be associated with the differentiation ability of cells, but not necessarily their proliferative capacity, proposing α2–6 Sia as a marker of the differentiation potential of hMSCs and primary chondrocytes. The group also reported a reduced differentiation efficiency in bone marrow-derived hMSCs in response to sialidase treatment, indicating that cell surface sialylation may play a functionally important role in the efficient differentiation of hMSCs [3].

The abundance of GAGs on the cell surface also varies significantly between pluripotent and differentiated cells. For example, murine embryonic stem cells (mESCs) exhibit low levels of surface GAGs. However, upon differentiation, levels of hyaluronan, chondroitin sulfate/dermatan sulfate, and heparan sulfate (HS) significantly increase. This is likely due to an increase in the expression of GAGs biosynthetic enzymes and core proteins [66]. Furthermore, GAGs display distinct sulfation patterns upon differentiation, indicating dynamic structural changes during embryonic development [67]. Sulfated glycans, including HS and CS, are known to regulate cell fate decisions, self-renewal, and pluripotency in stem cells by acting as co-receptors or stabilizing factors for signaling ligands. The latter includes Wnt, FGF, Hh, and BMP, which are central regulators of stemness, pluripotency, and differentiation fate decisions [1]. Furthermore, glycosylated cell adhesion molecules, including cadherins and integrins, play a vital role in the early development and maintenance of ESC [62,65].

In iPSC, HS stands out as the most prevalent GAG, constituting up to 80% of total GAG content, and its abundance differs significantly between pluripotent and differentiated cells [68]. Upon iPSC differentiation, HS content undergoes a remarkable increase, likely attributed to elevated transcript levels of HS biosynthetic enzymes and sulfotransferases. Similar trends were also observed in mESCs [66,67]. Lacking HS synthesis in mESCs leads to a pronounced differentiation defect, as these cells are unable to form any of the three germ layers and retain pluripotent markers [69,70,71]. This effect is likely due to aberrant cell signaling through FGF and Wnt pathways, both of which are modulated by direct HS binding to ligands in the extracellular space to facilitate signal transduction [72,73]. In aggregate, these findings strongly suggest that GAGs may serve as crucial regulators of cell fate decisions and pluripotency in stem cells.

As glycans can serve as markers for pluripotency and stem cell differentiation, monoclonal antibodies have been employed to tag and identify pluripotent cells by recognizing glycosylated transmembrane proteins [74]. These antigens include TRA–1–60 and TRA–1–81, which are heavily glycosylated podocalyxins and are expected to downregulate through the differentiation process [75]. In addition, α1–2–fucosylated glycans help distinguish pluripotent cells from differentiated cells. Other markers of stem cells including SSEA-5 and rBC2LCN recognize fucosylated glycan structures. rBC2LCN (recombinant N-terminal domain of the BC2L-C lectin from *Burkholderia ceno-cepacia*) is a well-known stem cell marker probe that selectively binds undifferentiated human iPS cells and ES cells, but not differentiated somatic cells [76]. The binding targets of rBC2LCN include Fuc1α1-2Galβ1-3GlcNAc (GalNAc)-containing glycans, such as H type1, H type3, Lewis b, and Globo H. Studies have additionally revealed that podocalyxin, a heavily glycosylated type 1 transmembrane protein, acts as a glycoprotein ligand for rBC2LCN on human iPS cells and ES cells [77]. This suggests a potential role for fucosylation in the maintenance of undifferentiated cells.

### 3.2. Central Nervous System

In Central Nervous Systems (CNS), N-glycosylation can be associated with neuronal development, differentiation, and regeneration. Sequential proteomic analysis composed of LC/MS/MS of tryptic digest, enriched glycopeptides, and deglycosylated peptides of proteins derived from iPSC and iPSC-derived neuronal cells, revealed that the glycosylation profiles were dynamically changed at each glycosylation site and in each glycoprotein during neuronal differentiation. Particularly, the levels of glycoproteins modified with an N-glycan, consisting of five HexNAc, three Hex, and a Fuc (HN5H3F), increased in dopaminergic neuron-rich cells. The HN5H3F-modified proteins were considered to be involved in neural cell adhesion, axon guidance, and the semaphorinplexin signaling pathway, and the observed protein modifications were protein, site, and differentiation selective regardless of protein production levels. Collectively, this data underscores the pivotal involvement of N-glycosylated proteins in the complex process of neuronal differentiation of iPSC [78]. Furthermore, an elevated abundance of sialic acid is evident in neuronal cell membranes when compared to other tissues [79]. The interactions of polysialic acid with different neurotrophic factors are involved in synaptic plasticity and neurogenesis. Additionally, the role of polysialic-neural cell adhesion molecule assumes prominence as a pivotal neuroplastic molecule crucial for memory formation, while a decline in polysialic acid emerges as a critical factor in the pathogenesis of schizophrenic brains [80].

### 3.3. Adipose Tissue

The N-glycome of bone morrow-derived MSCs during adipogenic differentiation was determined using matrix-assisted laser desorption ionization time-of-flight mass spectrometry combined with exoglycosidase digestions [81]. This approach unveiled a diverse array of more than 100 distinct N-glycan structures, encompassing high mannose, hybrid, complex N-glycans, along with poly-N-acetyllactosamine chains. Notably, a prominent trend emerged: adipogenesis correlated with heightened sialylation and biantennary fucosylated structures, concomitant with a reduction in fucosylated and fucosylated tri- and tetra-antennary structures. Cell markers exhibit a crucial role in affirming the homogeneity of MSC cultures and their differentiated adipogenic progeny. These markers facilitate continuous tracking of the progress of MSC development throughout adipogenesis and enable the meticulous analysis of key quality parameters, such as the distribution of cells within newly formed adipose tissue. Interestingly, specific N-glycans such as H6N5F1 and H7N6F1 exhibited substantial overexpression in undifferentiated MSCs, while H3N4F1 and H5N4F3 were found to be upregulated in adipogenically differentiated MSCs [81]. As a result, H6N5F1 and H7N6F1 were proposed as candidate markers for undifferentiated MSCs, while H3N4F1 and H5N4F3 were considered markers for identifying adipogenic differentiation of MSCs. Furthermore, Liu et al. (2018) investigated the dynamics of protein glycosylation during adipogenesis in ESCs, using the lectin microarray approach. Their findings revealed an elevation in GlcNAc and α–1–2–fucosylation levels in ESCs undergoing adipogenesis. In contrast, the levels of α–1–6–fucosylation and α–1–6–mannosylation were decreased during adipogenesis, suggesting the potential utility of these glycan structures as stem cell markers throughout the differentiation process [82]. In summary, the importance of glycan structures as potential markers for comprehensively monitoring the progress of adipogenic development should be considered for meticulous exploration.

### 3.4. Cardiac Tissue

Analysis of cardiomyocytes glycome, derived from hPSC (CM–hPSC) showed that 62 glycans were unique to hPSC at the early stages of the differentiation (days 0 and 7), which disappeared from the glycome analysis of CM–hPSC by day 15 [75,83]. These glycans included β1,3-linked galactose, α2,6-linked sialic acid, and other complex fucosylation, with CM–hPSC exhibiting higher levels of α2,3–sialylation [75,83]. DNA microarray analysis showed that mutations of the genes responsible for glycogen regulation in cardiomyocytes could lead to alteration in the glycosylation profile of mature cardiomyocytes and impairment of ion channel function [81,84]. Ion channels play a crucial role In action potential propagation and muscle tissue contraction within the heart [83]. Mutations in the N-glycosylation patterns of potassium and sodium channels result in hyperpolarization of the external cell surface, leading to conditions like long QT syndrome [85]. The glycosylation profile of hPSC from patients with long QT syndrome exhibited similar rhythm abnormalities and showed an altered glycosylation profile of the potassium channels. Furthermore, alterations in the glycosylation profile of potassium-gated channels subunit family H increased cardiac tissue excitability to specific medications, including Sulfamethoxazole [85]. The sialylation process influences the modulation of voltage-gated sodium channels within the nodal tissue and between the cardiac chambers. Neuraminidase treatment of cardiac tissue, which removes sialic acid covalent bonding from glycosylated proteins, results in depolarization of ion channels and dysfunction in action potential and calcium channel function. Additionally, O-glycosylation facilitates atrial and ventricular β1-adrenergic receptor activity for downstream signaling pathway enhancement [83].

### 3.5. Epidermal Stem Cells

Epidermal stem cells (EpiSCs) play a crucial role in skin development, metabolism, and repair. These specialized cells are distributed in the basal layer of the epidermis and hair follicles, endowed with an extraordinary capacity for proliferation, maintaining epidermal homeostasis [86]. The decline in tissue regeneration and function associated with aging is often linked to impaired epidermal stem cell function, as they struggle to effectively interact with other cell types or the extracellular matrix within the skin [87,88,89]. While genetic and epigenetic changes have been extensively documented in aging skin [90,91,92,93,94], recent studies are shedding light on the significant involvement of glycans in regulating epidermal stem cell behavior. For instance, glycosylation patterns have been observed to undergo dynamic changes in murine EpiSCs during aging. A comprehensive lectin microarray analysis of glycan profiles in freshly isolated EpiSCs from young and old mice unveiled pronounced disparities between the two groups. The binding affinity of rHeltuba, a lectin known for its specificity to mannose residues, was notably reduced in old epidermal stem cells. Conversely, the binding affinity of rGal8N, a lectin with an affinity for α2-3 sialic acid residues, exhibited a marked increase. These glycan alterations were accompanied by an upregulation of sialyltransferase genes (*St3gal2* and *St6gal1*) and the mannosidase gene *Man1a* in old EpiSCs. Interestingly, the targeted modulation of cell surface glycans through the overexpression of these genes mirrored the aging glycan patterns and hindered the growth of primary keratinocytes, resulting in a significant reduction in the regenerative potential of epidermal stem cells in culture [95]. Consequently, this study highlights the age-related global alterations in cellular glycosylation patterns and their potential contribution to stem cell function. These glycan modifications may serve as molecular markers for aging, and further functional studies would undoubtedly advance both regenerative therapy and the diagnosis of skin aging.

Basal keratinocytes host the stem cell population responsible for epidermal regeneration [96]. Among O-glycan structures, core 1 O-glycans, rather than core 2 structures, are predominantly expressed in basal keratinocytes, playing a pivotal role in inherent epithelial functions, including cell–cell adhesion. The O-linked N-acetylgalactosamine modification is the most abundant type of O-glycans and is known to undergo characteristic changes during cell differentiation and maturation within stratified squamous epithelia. To probe the role of O-glycans in epithelial homeostasis, Dabelsteen et al. (2020) introduced the N/TERT–1 cell model. This model involves keratinocytes lacking elongated O-glycans due to targeted modification of the core 1 synthase gene (C1GALT1) or its chaperone, COSMC. The results of their study underscored that the absence of C1GALT1 and COSMC led to delayed differentiation and impaired cell–cell adhesion. This adhesion impairment was linked to protein kinase C (PKC) activity, as inhibition of PKC restored cell–cell adhesion in C1GALT1-deficient N/TERT–1 cells. These observations were further supported by RNA-seq analysis of keratinocytes with disrupted C1GALT1 and COSMC, which revealed diminished expression of differentiation markers alongside elevated levels of cellular stress markers. Conversely, the sole loss of branched core 2 O-glycans triggered a minor impact on supra-basal differentiation without affecting intercellular interactions [97]. In contrast, core 2 structures predominantly manifest in supra-basal cell layers, where they are believed to regulate non-endogenous functions, particularly interactions with immune cells [97]. These findings emphasize the critical role of O-glycans, particularly core 1 structure, in maintaining epithelial integrity and function.

In conclusion, the interplay of epidermal stem cells and glycans can be considered as a crucial determinant in skin health and aging. The emerging insights into glycan-mediated regulation of stem cell behavior open avenues for innovative approaches in both regenerative therapies and diagnostic strategies for skin aging.

### 3.6. Osteogenic Tissue

Functional modulation of early osteogenic differentiation steps in an immortalized mesenchymal stem cell (iMSC) line model has been demonstrated through N- and O-glycan processing [98]. Inhibiting N-glycan processing in iMSC altered the differentiation and enhanced the mineralization capacity of the osteoblasts. This impact of N-glycans on iMSC differentiation has been associated with the phosphoinositide–3–kinase (PI3K)/Akt pathway, primarily due to a decrease in Akt phosphorylation. Interestingly, the study by Wilson et al. (2018) unveiled that inhibition of PI3K during the initial 2 days of osteogenesis could mimic the outcomes of inhibiting N-glycan processing. Thus, glycan processing provides another layer of regulation that can modulate the functional outcome of cellular differentiation, which could be reflected in novel therapeutically appealing processes [98]. On the other hand, investigations into mESC deficient in both N-deacetylase and N-sulfotransferase 1 and 2, crucial enzymes in HS synthesis, revealed intriguing insights. While the osteogenic differentiation remained unaffected, a lag in differentiation potential within the adipogenic and neural lineages was observed. These observations further confirm the specialized role of glycans within stem cells [39].

### 3.7. Hematopoietic Differentiation

A pivotal role of HS synthesis in the differentiation potential of ESCs was demonstrated by Holley et al. (2011). ESCs originating from embryos with HS synthesis deficiency exhibited an intriguing incapacity to progress toward hematopoietic lineages. Instead, these cells maintained persistent expression of ESC markers during embryoid body culture. Interestingly, the introduction of heparin, a shorter and soluble form of HS, into the culture medium triggered the initiation of hematopoietic differentiation, underscoring the selective role of HS in this process. The influence of heparin treatment extended to the activation of key signaling pathways including BMP, Smad, and Wnt. Interestingly, the concentration of heparin played a decisive role in shaping the outcomes, where a higher concentration of heparin resulted in a dose-dependent inhibition of hematopoietic differentiation. This observation does not only emphasize the significance of GAGs in hematopoietic differentiation, but also underscores the delicate balance required in heparin dosage for orchestrating this process [99].

## 4. Harnessing Synthetic Glycans to Control Stem Cell Differentiation

As our understanding of the factors influencing stem cell behavior deepens, we gain greater capabilities to manipulate their abilities and maximize their therapeutic benefits. Among these factors, cell surface glycans are increasingly identified as co-regulators or stabilizers of growth factor signaling essential for stem cell fate decision [1,100]. Synthetic glycans emerge as a versatile toolset for studying and harnessing the complex mechanisms that govern stem cell fate determination, opening novel avenues within regenerative medicine and tissue engineering. Biologically functionalized, engineered materials have the capacity to influence stem cell behavior through a synergistic blend of biological, mechanical, and topographical cues.

While genetic approaches to manipulate the expression of glycosyltransferase genes are available, their utility in glycan engineering has limitations due to the combinatorial nature of glycan biosynthesis and the functional redundancy of glycosyltransferase genes [101]. Additionally, genetic transfection using viral vectors may cause unpredictable risks, and irreversible gene modifications may raise safety concerns for clinical applications. Moreover, not all cell types can adapt to genetic alteration without side effects, particularly in stem cells [102,103]. Therefore, biochemical and chemical strategies offer valuable complements to these genetic approaches, notably by enabling the introduction of unnatural functionalities, such as fluorophores, into cell surface glycans.

### 4.1. Biochemical and Chemical Strategies in Glycan Engineering

Numerous studies have explored the application of synthetic glycans to direct stem cell differentiation. One such example is the use of engineered glycans to drive the differentiation of mESCs towards the mesodermal lineage [104]. The transition of mESCs from their pluripotent state to mesodermal cell lineage is orchestrated by the growth factors FGF2 and BMP4, respectively [105]. BMP4, via the Smad protein signaling pathway, downregulates FGF and Wnt signaling, thereby suppressing neuroectoderm formation and promoting mesoderm formation [105,106]. Remarkably, both the extracellular matrix (ECM) and cellular glycans play significant co-regulatory roles in this process. HS has been identified as a class of glycans involved in spatially patterning growth factors and facilitating signal transduction at the cell surface [70,107]. Consequently, the precise chemical manipulation of HS activity within the cellular glycocalyx of stem cells presents a promising effective control of cellular differentiation.

Naticchia et al. (2018) reported a novel method to enhance differentiation, utilizing lipid-functionalized synthetic HS-mimetic glycopolymers. These synthetic glycans exhibited a dual affinity for both FGF2 and BMP4, facilitating the mesodermal differentiation of mESCs in embryoid body culture. These glycans were introduced into the plasma membrane of mutant mESCs deficient in exostosin 1 and 2 (Ext1/2) glycotransferases, which are responsible for HS biosynthesis by adding alternating N-acetylglucosamine (GlcNAc) and glucuronic acid (GlcA) residues to the growing polysaccharide chain [104]. Remodeling the glycocalyx of these mutant Ext1−/− mESCs showcased an increased association of BMP4 at the cell surface, leading to enhanced mesodermal differentiation through the associated MAPK and Smad signaling pathways [104]. This study demonstrated the feasibility of using synthetic glycans to engineer the glycocalyx of Ext1−/− mESCs within three-dimensional embryoid body structures, providing valuable insights into the complex mechanisms governing stem cell differentiation and fostering potential therapeutic advancements.

On the other hand, collagen–GAG scaffolds have emerged as promising tools for bone tissue engineering. Synthetic GAGs possess a few advantages over natural counterparts, offering structural homogeneity, purity, and controlled sulfation to circumvent limitations [108,109]. Farrell et al. (2006) exemplified this potential by utilizing a collagen–glycosaminoglycan scaffold to promote the differentiation of adult rat mesenchymal stem cells towards the osteogenic and chondrogenic lineages [110]. Cultivating these cells on the collagen–GAG scaffold combined with the addition of osteogenic factors (dexamethasone, ascorbic acid, and beta-glycerophosphate) induced osteogenesis, as evident by the temporal induction of the bone-specific proteins, collagen I and osteocalcin, as well as subsequent matrix mineralization and the activation of the extracellular-regulated protein kinase (ERK), which is involved in the osteogenic process. Conversely, exposing the scaffold-seeded cells to chondrogenic factors, dexamethasone and transforming growth factor–1 beta, enhanced collagen II immunoreactivity, suggesting that the scaffold can be used to generate a suitable three-dimensional environment that supports chondrogenesis [110].

Sulfated glycosaminoglycans play pivotal roles in regulating stem cell lineage commitment and differentiation within the bone marrow stem cell niche and mature bone tissue. An interesting study by Hempel et al. (2014) provided valuable insights into the utilization of artificial extracellular matrices (aECMs) as influential factors in shaping the differentiation of osteoblast precursor cells and early osteoblasts. This investigation’s premise involved the preparation of aECMs through the gradual sulfation of chondroitin sulfate and hyaluronan derivatives [111]. Human bone marrow stromal cells were used to identify the most potent aECM formulation that drives pro-osteogenic effects, as evaluated by the influence of sulfate groups, as well as the type of disaccharide integrated into aECM. The results of the study revealed that over-sulfated GAG derivatives, characterized by a sulfate group positioned at the C–6 site of N-acetylglycosamine, exhibited the most pronounced and effective pro-osteogenic impact, as evaluated by tissue nonspecific alkaline phosphatase activity and calcium deposition. Subsequent analysis encompassing a subset of aECMs in association with primary osteoblasts and cell lines representing diverse maturation stages reaffirmed the notable pro-osteogenic influence specifically on early osteoblasts [111]. Through a comprehensive approach that encompasses molecular positioning, structure, and biological response, this study highlights the significance of over-sulfated GAG derivatives as influential players in steering early osteoblast differentiation. The findings underscore the potential of tailored aECMs in modulating stem cell behavior within their niche, thereby advancing our understanding of osteogenesis.

Apart from osteogenic differentiation, synthetic GAGs exhibit a remarkable potential to induce neural differentiation. Wang et al. (2015) introduced an innovative strategy to prepare GAGs analogs by splitting and recombining sulfated saccharide units found in natural GAGs. They employed monomers (SS and MAG) containing essential GAG structural units as building blocks to synthesize polymers with well-defined chemical structures and adjustable ratios of functional units through living radical polymerization [108]. The synthetic polymers exhibited robust bioactivity, promoting both cell proliferation and neural differentiation of ESCs. The results of the study further revealed distinct roles played by unit S and unit G in influencing GAG bioactivities. Significantly, these synthetic polymers demonstrated superior bioactivity compared to heparin, highlighting their potential to enhance our comprehension of biomacromolecule structure–function relationships and create alternatives to complex natural macromolecules [108].

Furthermore, novel strategies in cell surface engineering have harnessed the function of HS, which mediates interactions between growth factors and their receptors, to promote the differentiation of ESCs. In an interesting example, Huang et al. (2014) generated a synthetic neoproteoglycan with an affinity for FGF2 and integrated it into the plasma membrane of HS-deficient ESCs to induce neuronal differentiation. The study revealed that neoproteoglycan retained the function of native HS, effectively rescuing FGF2 activity and promoting neural specification, which demonstrates the versatility of glycocalyx remodeling for potential application in diverse differentiation processes [112]. Another innovative approach involved the functionalization of electrospun scaffolds with GAGs through ionic immobilization onto fiber surfaces [113]. This binding strategy preserved GAGs’ interaction capability with binding molecules and showcased essential GAG sulfation motifs pivotal for orchestrating stem cell behavior. These GAGs successfully rescued the neural differentiation capacity of HS-deficient mESCs and, in synergy with FGF4, facilitated extensive neural process formation across the scaffold surface [113]. The combination of GAGs with electrospun scaffolds establishes a potent biomaterial platform for stem cell propagation and differentiation, holding great promise for tissue engineering and regenerative medicine applications.

Collectively, these studies underscore the potential of synthetic glycans in driving stem cell differentiation and suggest a foundation for tailored stem cell differentiation strategies with promising therapeutic applications. Continued investigations and refinements in the design and application of synthetic glycans will undoubtedly lead to even greater advancements in the field of stem cell-based therapies, potentially revolutionizing the treatment of various medical conditions.

### 4.2. Metabolic Glycoengineering

Metabolic glycoengineering (MGE) is another approach that is being exploited to control stem cell activities. Although MGE technique was introduced over three decades ago [114], it is currently finding renewed interest in modeling stem cell niches and controlling their fate [101]. The primary goal of MGE is to augment the expression of natural glycans and incorporate non-natural monosaccharides into cell surface glycoconjugates, such as ketone-, azide-, thiol-, or alkyne-modified glycans [115,116].

Since MGE exploits the innate metabolic pathway of cells, the modification process minimally disrupts other cellular functions [117]. Additionally, the MGE strategy possesses several distinctive advantages, being an easy, yet highly efficient process, and achieved through straightforward coculturing of cells with metabolic precursors. Remarkably, MGE exhibits no cytotoxicity even under high treatment concentration and its applicability extends to nearly all cell types, rendering it a versatile tool in the field. Furthermore, the modifications introduced by MGE are nonpermanent, enabling controlled reversibility. Bioorthogonal click chemistry and the wide array of sugar analogs available further contribute to the versatility of MGE by offering diverse options for membrane modification [101].

**Figure 1:**
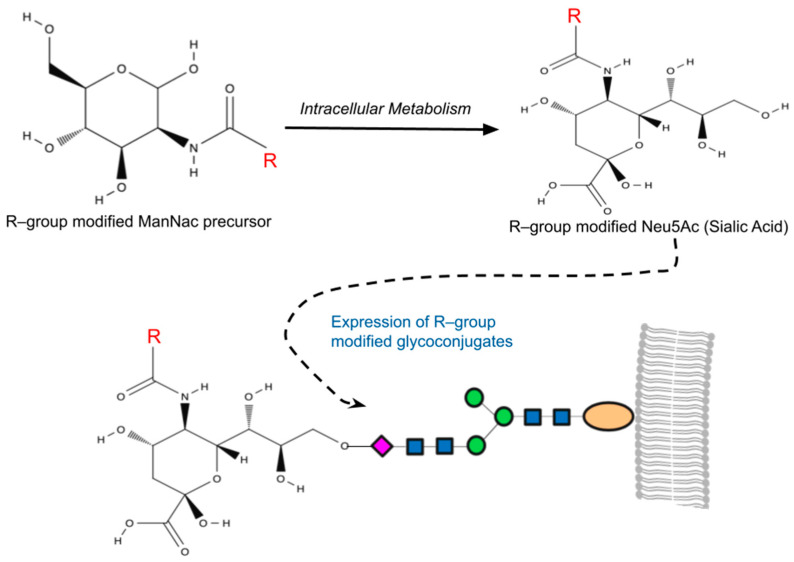
A simplified overview of metabolic glycoengineering (MGE). MGE involves the introduction of diverse chemical groups into cellular glycans through artificially modified monosaccharides containing unnatural functionalities (R-groups). Mammalian cells incubated with the R-group-modified N-Acetylmannosamines (ManNAc) metabolize these precursors intracellularly, resulting in the production of non-natural sialic acids (Neu5Ac). This process leads to the presentation of R-group-modified glycans on cell surfaces or their secretion as glycoconjugates.

The sialic acid pathway was the first glycosylation pathway harnessed in MGE [118], and it remains the most frequently utilized pathway to date. The suitability of the sialic acid pathway for MGE lies in the notable substrate versatility of sialyltransferases [119], which enables the modified analogs to effectively intercept glycosylation pathways, resulting in chemically altered sialic [101]. Among human cells, N-Acetylneuraminic acid (Neu5Ac) is the most common form of sialic acid, while N-acetyl–D–mannosamine (ManNAc) serves as the physiological precursor of all sialic acids. Once internalized as a precursor within a cell, ManNAc undergoes conversion to Neu5Ac with the help of specific sialyltransferases, ultimately becoming anchored to the residues of cell surface sialic acid (Figure 1) [120].

Numerous studies have highlighted the applicability of MGE analogs in modulating stem cell behavior. For example, pretreatment of peracetylated N-thiolglycolyl–d–mannosamine (Ac5ManNTGc), a hyperacetylated ManNAc analog with a thiol group on its N-acyl side chain, significantly enhanced the adhesion capabilities of Jurkat cells—a property previously absent in this T–lymphoma-derived cell line [121]. This pretreatment also induced their expression of ECM components and upregulated the expression of β1–integrin, MMP–9, and CD44 [122]. Beyond adhesion, MGE extends its influence on cellular differentiation. Notably, the application of Ac5ManNTGc was shown to promote neural lineage differentiation in human embryoid body-derived (hEBD) stem cells, even in the absence of Wnt signaling proteins that are essential for neural differentiation [121,123]. Noteworthily, Wnt pathway upregulation and the response of Jurkat cells to Ac5ManNTGc treatment were scaffold dependent, occurring only when the cells were cultured on gold- or maleimide-covered surfaces where the thiol-modified cell surface sialic acids could form high-affinity bonds with the substrate. While scientifically intriguing, this approach faced limitations for translational research due to the challenges associated with developing in vivo applications dependent on a gold-plated surface or other high-affinity scaffolds. To address this issue, Du et al. (2021) developed two novel thiolated analogs, namely Ac5ManNTProp and Ac5ManNTBut, which install thiol on an elongated N-acyl side chain, effectively substituting natural cell surface sialic acids with their thiolated counterparts [124]. Treatment of human neural stem cells (hNSCs) with these thiolated analogs enhanced the ability of glycans to interact with naturally occurring endogenous thiols present in the cellular nano and microenvironment. This, in turn, enhanced the differentiation of hNSCs as well as their adhesion to extracellular matrix components in the absence of a complementary high-affinity scaffold [124]. Thereby, advancing the in vivo applications and potentially paving the way for clinical translation of these MGE analogs. Building on the previous studies, the group further expanded the applications of thiol-modified MGE analogs by demonstrating the ability of Ac5ManNTProp (tProp) to facilitate Schwann cells (SCs) differentiation from Adipose-derived stem cells (ASCs) [125]. SCs are myelinating cells essential for peripheral nerve regeneration [126]. SCs are often depleted when nerve lesions occur, hindering the repair process [127]. Addressing the limited and slow expansion capacity of SCs, ASCs have emerged as a promising therapeutic avenue for peripheral nerve injuries [128]. While ASCs possess SC differentiation potential, their natural transdifferentiation period exceeds two weeks [129]. To overcome this limitation, Du et al. (2023) harnessed MGE technology to expedite ASC differentiation into SCs. Specifically, the sugar analog tProp significantly enhanced ASC differentiation, leading to elevated expression of SC proteins S100β and p75NGFR, along with heightened levels of neurotrophic factors such as nerve growth factor beta (NGFβ) and glial cell-line-derived neurotrophic factor (GDNF). Remarkably, tProp treatment reduced the SC transdifferentiation period from approximately two weeks to just two days in vitro [125]. This breakthrough holds the potential to significantly improve neuronal regeneration.

Beyond sialic acid, additional glycosylation pathways have been harnessed in MGE. In a seminal study, Sackstein et al. (2008) demonstrated the profound impact of introducing fucose to cell-surface glycoprotein receptors in enhancing the trafficking of mesenchymal stem cells (MSCs) to bone. The group addressed a critical limitation in the clinical effectiveness of MSCs, which show promise in treating skeletal diseases but suffer from poor homing to bone [130]. The recruitment of cells to bone takes place within specialized marrow vessels expressing vascular E–selectin, a lectin that recognizes sialofucosylated determinants on its ligands [131,132]. Notably, it was observed that human MSCs lack E–selectin ligands but instead express a CD44 glycoform bearing alpha–2,3–sialyl modifications [133]. Through glycan engineering using an alpha–1,3–fucosyltransferase preparation, the research team successfully fucosylated the native CD44 glycoform on MSCs, transforming it into a hematopoietic cell E–selectin/L–selectin ligand (HCELL). This modification enhanced E–selectin binding without compromising cell viability or multipotency. Real-time intravital microscopy in immunocompromised mice revealed that intravenously infused HCELL(+) MSCs swiftly infiltrated marrow, leading to rare foci of endosteally localized cells and the generation of human osteoid [130]. This innovative approach not only provided a blueprint for programming cellular trafficking, but also underscored the broader potential of glycan engineering, particularly fucosylation, in directing the homing of various stem cell types to specific tissues. The study marks a significant leap forward in the field, offering a promising strategy for advancing stem cell-based therapies, not only for skeletal diseases but also for broader applications.

Overall, the aforementioned advances in MGE demonstrate its potential in providing fine-tuned control over stem cell fate as well as opening new avenues for the study of cellular niches and developmental pathways. Continued research and refinement in synthetic glycans and metabolic glycoengineering will undoubtedly lead to greater advancements in the field, ushering in a new era of tailored stem cell differentiation strategies with broad therapeutic applications.

## 5. Conclusions

The importance of glycans could have been underestimated in the world of stem cells. Although different glycans were considered as cell surface markers for stem cell characterization and sorting, their role may expand to include the response of the cells to differentiation mediators, as well as direct induction of intracellular signaling cascades. Furthermore, quantitative and qualitative analysis of different glycans can help in the identification of the cell differentiation status. From a bioengineering perspective, these properties can be used to enhance differentiation by providing the corresponding glycans in the cell culture environment. Because of the nature of these glycans, they can be easily incorporated into three-dimensional scaffolds to enhance the interaction with the stem cells as well as their differentiation. Thus, the role of glycans should be considered in stem cell differentiation programs as an additional factor to our current protocols. Further studies are required to describe the mechanistic details involved in the induction of pluripotency as well as differentiation. Other challenges include in vivo confirmation of the in vitro data as well as the determination of the biological effect of multiple glycosylation on stem cell fate.

## Figures and Tables

**Table 1:** Examples of glycosylation use/potential use as markers in relation to stem cells and their differentiation.

Glycosylation Marker	Use/Potential Use
α–1–2–fucosylation	Adipogenic differentiation of ESC
α–1–6–fucosylation	Adipogenic differentiation of ESC
α–1–6–mannosylation	Adipogenic differentiation of ESC
α2–6 Sia	Chondrocyte differentiation of hMSC
GlcNAc	Adipogenic differentiation of ESC
H3N4F1	Adipogenic differentiation
H5N4F3	Adipogenic differentiation
H6N5F1	Undifferentiated MSCs
H7N6F1	Undifferentiated MSCs
rBC2LCN	Marker of stemness
SSEA-5	Marker of stemness

H: Hex, N: HexNAc, F: Fuc.

**Table 2:** Glycosylation and its effect on stem cells and their differentiation.

Tissue	Glycosylation Effect
Adipose tissue	H3N4F1 and H5N4F3 N-glycans upregulated in MSCs GlcNAc and α–1–2–fucosylation increased in ESCs α–1–6–fucosylation and α–1–6–mannosylation decreased
Cardiac tissue	β1,3-linked galactose and α2,6-linked sialic acid and other fucosylaiomes in CM–hPSC early differentiation CM–hPSC exhibits high levels of α2,3–sialylation O-glycosylation controls β1–adrenergic receptor downstream signaling N-glycosylation facilitates potassium and sodium channels’ function
Central nervous system	GD1a ganglioside marker of neuronal differentiation High levels of polysialic acid lead to synaptic growth and regeneration Sustained exposure to high levels leads to decreased myelination CS and DS regulate morphogenesis, proliferation and differentiation, and CNS development HN5H3F-modified proteins involved in neural cell differentiation, adhesion, axon guidance, and semaphorin–plexin signaling pathway Polysialylation is involved in brain plasticity and neurogenesis and is highly expressed in neuroblastic and Schwann cells UDP–GlcNAc 2–epimerase activity increases with maturation in brain tissue
Epidermis	CS regulates wound repair Knocking out C1GALT1 and COSMC delays differentiation and compromised cell–cell adhesion through PKC pathway Core–1 O-glycans primarily expressed in basal keratinocytes and are essential for their functions
Hematopoiesis	HS-deficient ESCs are unable to differentiate into hematopoietic lineages
Naïve stem cells	O-glycosyltransferases increase in embryoid bodies compared to ESC O-glycosylation maintains epithelial state of trophoblast stem cells hPSCs exhibit high levels of α2–6-linked sialic acid mESC display low surface GAGs, while hyaluronan, CS, DS, and HS increase with differentiation HS and DS are markers of pluripotency by acting as coreceptors or stabilizing factors for signaling ligands Lack of HS synthesis decreases differentiation of mESC potential and maintains pluripotency iPSC has higher levels of surface α2–6-linked sialic acid, compared to differentiated progenitors Inhibition of α2,6–sialyltransferase leads to downregulation of OCT4 Hyperexpression of α2,6–sialyltransferase leads to pluripotent stem cell lineage commitment and reduced pluripotency Sialic acid removal from iPS–MBMC resulted in high β–galactopyranoside and differentiation Binding of α2–6 Sia-specific lectins is associated with cellular differentiation
Osteogenic tissue	Inhibiting N-glycan processing in iMSC enhanced mineralization of osteoblasts mediated by (PI3K)/Akt pathway

CNS: Central Nervous System, CS: Chondroitin Sulfate, C1GALT1: Core–1 Synthase Gene, CM–hPSC: Cardiomyocytes Derived Human Pluripotent Stem Cells, DS: Dermatan Sulfate, ESC: Embryonic Stem Cells, mESC: Murine Embryonic Stem Cells, iMSC: Immortalized Mesenchymal Stem Cell, iPSC: Induced Pluripotent Stem Cells, iPS–MBMC: iPS generated from Menstrual Blood-derived Mesenchymal Cells, PKC: Protein Kinase C, PI3K/Akt: Phosphoinositide–3–Kinase/Akt Pathway.

## Data Availability

Not applicable.
